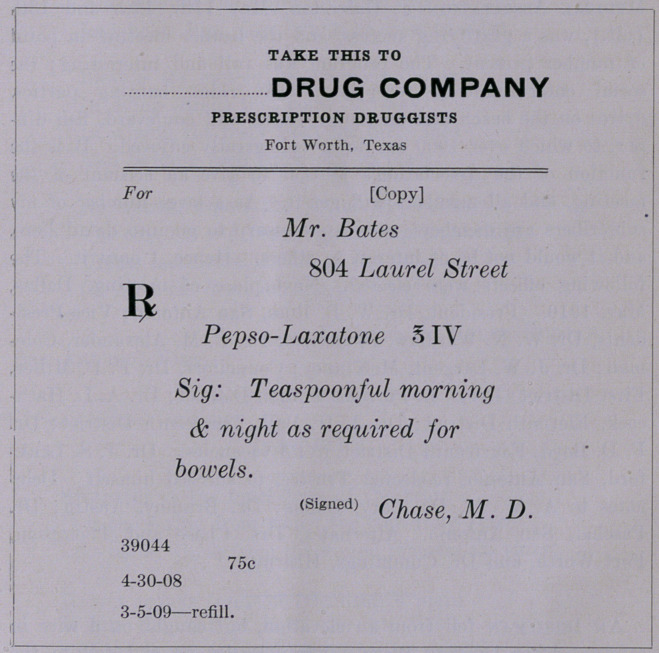# The Passing of Aristocracy in Medicine

**Published:** 1909-06

**Authors:** 


### EDITORIAL DEPARTMENT.

### THE PASSING OF ARISTOCRACY IN MEDICINE.

*"I have fought a good fight. I have finished my course. I have kept the faith."* 4 Timothy, 7.

*"Oh Jerusalem, Jerusalem, thou that killeth the prophets and stoneth them which are sent unto thee,—how often would I have gathered thy children together, even as a hen gathereth her brood under her wings, but ye would not."* 24 Matthew, 37.

The illustrious founders of the Texas State Medical Association made it an Association of Medical Aristocrats. It was composed of Southern gentlemen. It was exclusive. Its portals were guarded with jealous care, that none of even questionable ethics should be admitted. All old members remember how Dr. Ross was tried for serving on a local board with a homeopath; and how, a few years ago, a resolution was unanimously adopted to "expel any member who should so lower the dignity of his profession as to consult with a homeopath." Now the doors are thrown open to everything called "doctor," and all the irregulars and quacks and negroes are not only eligible to membership, but are invited and urged, through the columns of the Association's journal, to come in and affiliate. (See January number, page 216.) The McCormack Constitution under which we are operating throws the doors open to "the entire medical profession of Texas," and Chase bids them come. The A. M. A. makes no distinction whatever, and even negroes are members. The doctrine of "Brotherhood in Medicine" and "Unification" has been accepted by those in control in Texas, and no voice in opposition will be listened to in the House of Delegates; the rank and file have none. The revolution seems complete.

But, the TEXAS MEDICAL JOURNAL, which for twenty odd years opposed the tendency quackwards—the degradation of the profession to the level of the sectarians—will make one more stand to stem the downward rush; then, if the Daniel amendment fails at Dallas next May, farewell, a long farewell, to the old rule of aristocracy—the domination of rational medicine.

But it will not fail! The body of the membership are entitled to a voice, and they shall have it. The amendment limits membership to *white men of the regular profession, only,* and I feel confident of the support of all self-respecting Southern physicians. The voice of the "Red Back" was stifled in the House of Delegates; gag-rule and ignorance of parliamentary law prevailed, and the Big Chauffeur (on the bridge), with the President (on the back seat), ran the machine over all opposition.

Like Mirzah, I had a vision. I dozed in my seat, returning to Austin. I saw a convention of doctors—of many kinds. I saw a big man and a small man presiding. I heard a babel of voices. I saw a gray-haired man—of the old school—attempt to protest against defamation, falsehood and libel in the Association's journal. He was told to shut up. I saw hovering over the assembly the shades of seven Wise Men in solemn procession, with downcast eyes and averted faces. I saw their gray hairs and their celestial robes glint and gleam in the sunlight of a May morning. My spiritual eyes recognized the Illustrious Fathers who have passed to their reward, and dwell now in the Empyrean spheres in Immortality—Ashbel Smith, Starley, Glorious Swearingen, Cupples, Becton, Hadra—led by the Patriarch Nathan Smith Davis, and I lifted up my voice: "Oh ye Shades of Departed Greatness,—Apotheoses of Scientific Medicine! Save us, save thy heritage from the levelers!"

The scene shifted. I saw a red *Machine* coming down the road —its great eyes shone with demoniacal light. The chauffeur was a big man. I was in the way. I made out the name of the Machine. It spelled "Octopus, Jr." I was rattled and stood still, and was knocked sky-high. There was a "bump, bump," and all was still. The brakeman called out:

"Hempstead! Change cars for Austin."

Reminiscent—A Bit of Ancient History.—In the 80's the medical profession of New York split on the question of consulting with homeopaths. A large number of distinguished men seceded and organized the Kings County Medical Society, and adopted a code recognizing homeopaths. They became known as "The New Coders." The American Medical Association (N. S. Davis, President) met in Milwaukee (1884). Delegates from the New Coders were refused seats. The American Medical Association appointed a committee, with J. S. Billings, Chairman, to go to Copenhagen, Denmark, to the International Medical Convention, and invite the Congress to meet in America in 1885. Many New Coders went. These threatened Billings' committee that unless given recognition, in organizing the Congress, they would defeat the invitation. The committee agreed to the terms, and the invitation to meet in America was accepted. They came, and met in Washington. Billings, to keep faith with the New Coders, proceeded to organize the Congress—he and his committee—and appointed all the New Coders to prominent positions, ignoring the South and West entirely. One from New Orleans—and one man from Texas—alone were mentioned! This was usurpation of the power that belonged solely to the A. M. A., whose committee they were. The A. M. A. met in New Orleans in April, 1885. A large Texas delegation went. Drs. Shoemaker, of Philadelphia; Garnett, of Washington; Keller, of Hot Springs; X. O. Scott, of Ohio; Daniel, of Texas, and others, held a caucus and arranged a program to defeat the Billings crowd and the New Coders. Campbell, of Georgia, was presiding. The committee (thro' Billings), with unparalleled assurance, presented the report of their organization of the Congress, reading out the names of all officers, including Jacobi and other New Coders, who had been repudiated and refused recognition by the A. M. A. at Milwaukee! Shoemaker had arranged to be recognized first. He took the floor and in a scathing speech exposed the trick. Daniel was next recognized (there were fifty on their feet calling "Mr. President"). Dr. Daniel protested against the organization of the Congress by this committee and especially against the injustice done the South and the West in ignoring the medical men of those sections, holding that the organization of the Congress devolved upon the Association, and that the only thing required of the committee was to report back to the Association that they had discharged their duty—extended the invitation—and that it had been accepted. Dr. Daniel then moved that the report be not *received;* and that a Committee on Organization be now appointed, consisting of one member from each State, to organize the Congress. This was carried. The Billings committee was discharged from further consideration of the subject. Our Dr. J. W. McLaughlin represented Texas. The committee met in St. Louis, organized the Congress, and the South and West received recognition, Texas getting five offices—Swearingen, Paine, Cupples, McLaughlin and Daniel. Meantime the disgruntled (New Coders) went to law to get an injunction or something, but were advised by Speaker Randall of Congress that Daniel was right in his position, that the organization of the Congress devolved on the Association and not on the committee; and that the action at New Orleans was in accordance with parliamentary law, that a committee could not rise above the source of its appointing.

Then followed a war in the medical press, Shrady of the *New York Medical Record* leading the New Code forces, and Daniel of the TEXAS MEDICAL JOURNAL championing the cause of the South and West. We whipped them out completely.

The "Red Back" then epitomized the event, and immortalized it by the following parody:

THE DESTRUCTION OF SHENANEGAN.

(*After Byron: "The Destruction of Sennacherib."*)

The Chairman came forward so gallant and bold,
And his shirt-front was gleaming with diamonds and gold;
The smile on the faces was fearful to see,
As he read the report of that deep commit-tee.
The appointments they made were like the sands on the shore;—
They gave all to their friends—and would gladly give more,—
Ignoring the members from the West and the South,
Which brought forth destruction from Shoemaker's mouth.
He told of the bargains with New Coders made—
The plans and maneuvers that Jacobi laid
To capture the Congress in this mighty nation,
By an extra sharp game on the *As*-so-ci-ation!
Then the fire from their eyes, like a sword from its sheath,
Flashed forth in its vengeance; and gnashing their teeth,
They glared like a demon on the men from the South,
And the impotent froth-foam rose white on the mouth!
But the Angel of Justice spread his wings on the blast,
And whispered "No go," to those men as he passed.
Then the thought of the schemers was like the poison in the cup;
Because thus defeated they'd "bust the thing up!"
And the Doctors of 'Delphia are loud in their wail,
Because right, and justice, and *ethics* prevail.
Thus the game of Shenanegan the profession soon saw
Would never "hold water," in the eyes of the law.

---

THE JEWEL CONSISTENCY.—Speaking of uncensored proprietaries; i. e., those that have not been approved by the A. M. A. Council of Pharmacy; those that have been so bitterly denounced by the editor of the *Texas State Journal of Medicine,* Chase, and for prescribing which physicians have been denounced, the following will be of interest:

American Medical Association
Council on Pharmacy and Chemistry.
103 Dearborn Ave., Chicago, April 26, 1909.

*Dr. F. E. Daniel, Editor the Texas Medical Journal, Austin, Texas.*

Dear Sir: The product (Pepso Laxatone) in regard to which you inquire in your letter of April 23d has not been considered by the Council.

Very truly yours,
W. A. Buckner,
Secretary.

**TAKE THIS TO**

**..................DRUG COMPANY**

**PRESCRIPTION DRUGGISTS**

Fort Worth, Texas

*For* [Copy]

*Mr. Bates*

804 *Laurel Street*

℞

*Pepso-Laxatone* ʒ IV

*Sig: Teaspoonful morning*
*& night as required for*
*bowels.*

(Signed) *Chase, M. D.*

39044
75c
4-30-08
3-5-09—refill.

State Board of Health.—The Governor has appointed the following named physicians to be the State Board of Health provided for by the Thirty-first Legislature: Dr. W. M. Brumby, President, and to be called "State Health Officer"; Dr. H. W.

Cummings, of Hearne; Dr. J. E. Gilcreest, of Gainesville; Dr. Boyd Cornick, San Angelo; Dr. J. E. Burns, Cuero; Dr. M. H. E. Whitesides, of Timpson, and Dr. T. F. Burnet, of Seymour, Texas.

The Board met in Austin May 25th, and organized. They appointed Dr. L. B. Bibb, Registrar of Vital Statistics; Dr. E. H. Lancaster, Bacteriologist and Chemist; Dr. T. U. Painter, Assistant State Health Officer, and J. N. Wilkins, Sanitary Inspector, but, as the appropriation for the last two offices was cut out by the Governor, they are dead.

---

The Forty-first Annual Convention of the Texas State Medical Association at Galveston, May 11th, 12th and 13th (ult.), was a gratifying success, and the banner meeting in point of number present. The program was full and interesting; the social entertainments—receptions, boat rides, bathing parties, drives on the beach and on the great sea wall boulevard, fish dinner, to which over 1000 sat down—all greatly enjoyed. It is the function of the Association's journal to give an account of the meeting, and all members will get it. As a large number of my subscribers are members, it is unnecessary to go into detail here, and it would not be of interest to others. Hence, I omit it. The following officers were elected. Next place of meeting, Dallas, May, 1910. President, Dr. W. B. Russ, San Antonio; Vice-Presidents, Dr. W. N. Wardlaw, Plainview; Dr. C. M. Alexander, Coleman; Dr. J. W. Largent, McKinney; Councilors, Dr. F. P. Miller, First District; Dr. S. E. Parsons, Fourth District; Dr. A. L. Hathcock, Eleventh District; Dr. J. H. Ball, Thirteenth District; Dr. F. D. Boyd, Fourteenth District, to fill vacancies. Dr. J. S. Lankford, San Antonio, re-elected Trustee, to succeed himself. Delegates to A. M. A., Dr. Cary, Dallas; Dr. Brumby, Austin; Dr. Paschal, San Antonio. Alternates, Drs. Chase and Thompson, Fort Worth, and Dr. Cummings, Hearne.

---

An Irishman fell from an elevation, but caught on a wire in safety. After hanging there a while he let go and fell to the ground, some twenty feet. A bystander asked:

"What made you let go the wire?"

"I was afraid the dom thing would break," said Pat.

His brother was painting a fence and was working very rapidly. "What are you in such a hurry for, Mike?" asked the spectator.

"I want to finish my job before the paint gives out," said Mike.

## Figures and Tables

**Figure f1:**